# Supplementary material for: Intermittent horizontal mattress suture in proximal anastomosis for acute type A aortic dissection: a retrospective study
Source: PeerJ. 2025 Mar 26;13:e19159. doi: 10.7717/peerj.19159 (PMC11954457; doi:10.7717/peerj.19159)
Supplement: Supplemental Information 1 [file peerj-13-19159-s001.docx]

**Operative technique**

**Cardiopulmonary bypass**

After the induction of general anesthesia and a median sternotomy, cardiopulmonary bypass was initiated using femoral artery cannulation for perfusion and vena cava cannulation for venous drainage. A left atrial vent was introduced into the left atrium via the right superior pulmonary vein. Myocardial protection was achieved by antegrade infusion of cold blood cardioplegia. A longitudinal incision of the ascending aorta was made between the brachiocephalic trunk and sinus-tubular junction.

**Total arch replacement and the frozen elephant trunk**

Circulatory arrest was implemented once moderate hypothermia was reached, with the bladder temperature maintained at 26–27 °C. Unilateral cerebral perfusion was achieved through selective cannulation of the left common carotid artery. Following longitudinal opening of the aortic arch, a stented elephant trunk was carefully inserted into the true lumen of the descending aorta. Complete coverage of the orifice of the left subclavian artery (LSA) by the stented elephant trunk was followed by external occlusion of the LSA root. Subsequently, the distal end of the four-branched artificial graft was anastomosed with the proximal end of the stented elephant trunk and aortic wall. Subsequently, systemic perfusion was re-established through the femoral artery cannula, with hemostasis meticulously maintained at the final anastomosis site. Finally, the LSA and brachiocephalic trunk were connected to the two 8-mm branches of the four-branched artificial graft using 5–0 polypropyleneProlene sutures, and bilateral cerebral perfusion was restored.

**Reinforcement of Fragile Aortic Wall**

In cases where the aortic wall at the aortic root is was fragile, we reinforced the weakened aortic wall by placing narrow strips of artificial blood vessel, with their inner surfaces facing inward, separately onto both the inner and outer layers of the aortic root. During the process of IHMS technique, sutures were passed through both the vascular strips and the aortic wall to prevent tearing.

**Double-patch sandwich technique for proximal anastomosis**

In this approach, Teflon felt strips were precisely situated both inside and outside the suture line, with a continuous transverse mattress suture using 4–0 polypropylene sutures to finalize the double-patch sandwich technique. Subsequently, continuous anastomosis of a segment of the artificial graft to the double-patch sandwich using 4–0 polypropylene sutures was performed. After anastomosis, cardioplegia was infused through the artificial graft to detect any potential leaks at the anastomosis site and evaluate aortic valvular function. The remainder of the procedure resembled that of the IHMS technique.

**The management methods of bleeding at the suture site of the aorta root following cardiac resuscitation is were the same for both the sandwich and IHMS techniques.**
