# Supplementary material for: Intermittent horizontal mattress suture in proximal anastomosis for acute type A aortic dissection: a retrospective study
Source: PeerJ. 2025 Mar 26;13:e19159. doi: 10.7717/peerj.19159 (PMC11954457; doi:10.7717/peerj.19159)

**Fig. S1.** The suture was secured with a suture anchoring coil device.


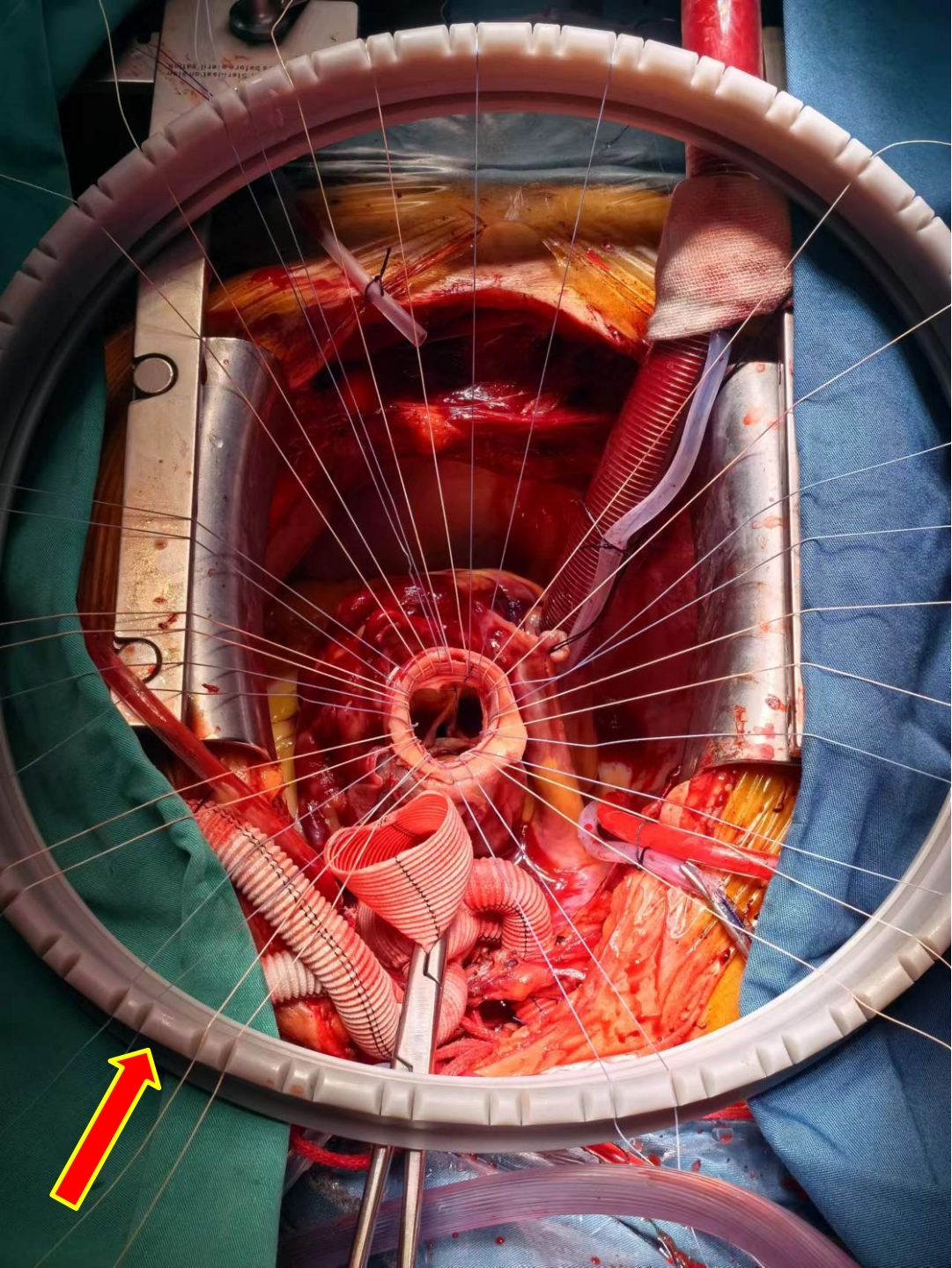


**Fig. S2.** The artificial vessel strips was lined with adventitia and intima.


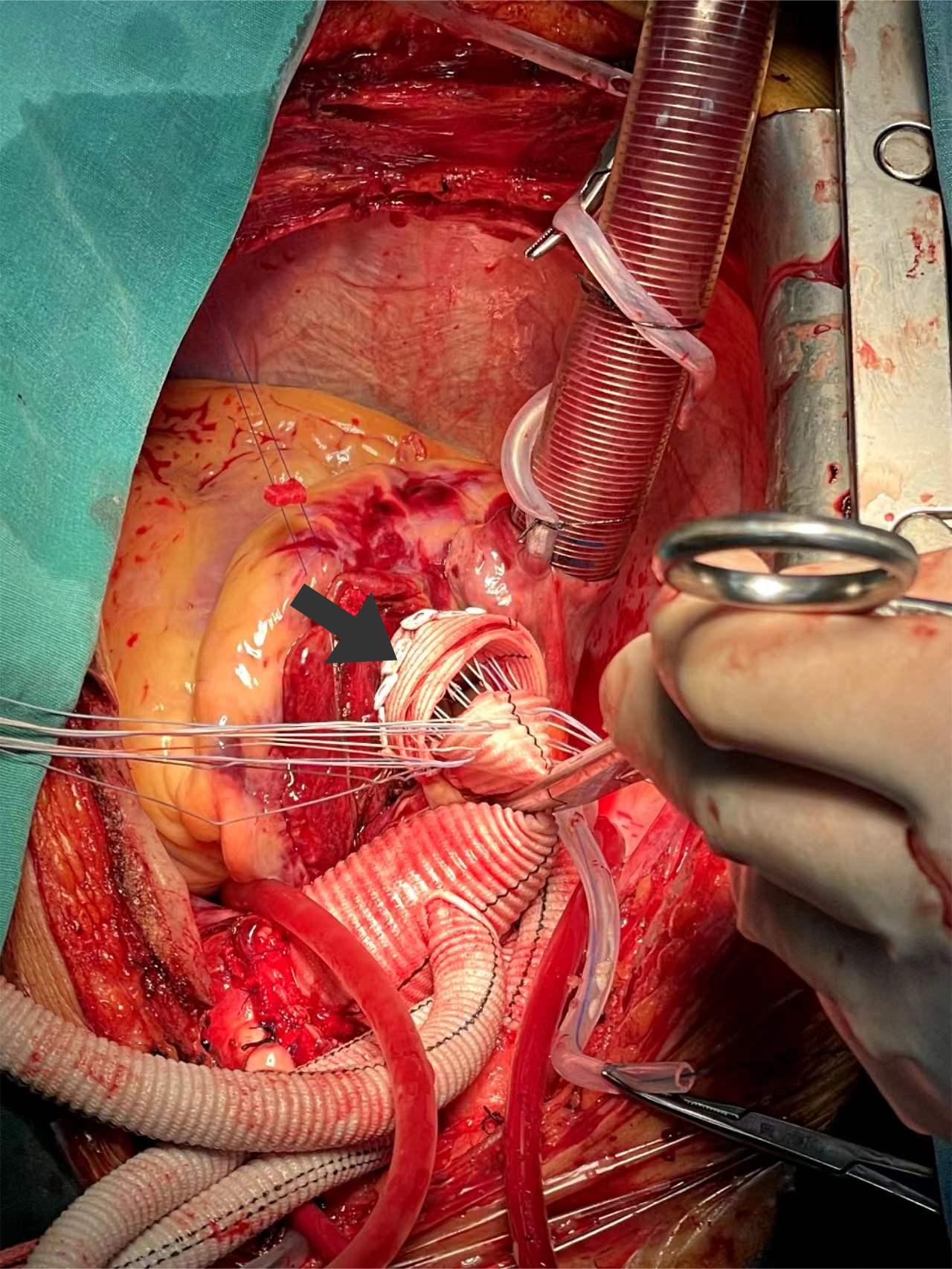


**Fig. S3.** Flow diagram for patient enrollment. ATAAD = acute type A aortic dissection; TAR = total arch replacement; FET = frozen elephant trunk; CABG = coronary artery bypass grafting; U-ACP = unilateral antegrade cerebral perfusion; IHMS = intermittent horizontal mattress sutures.


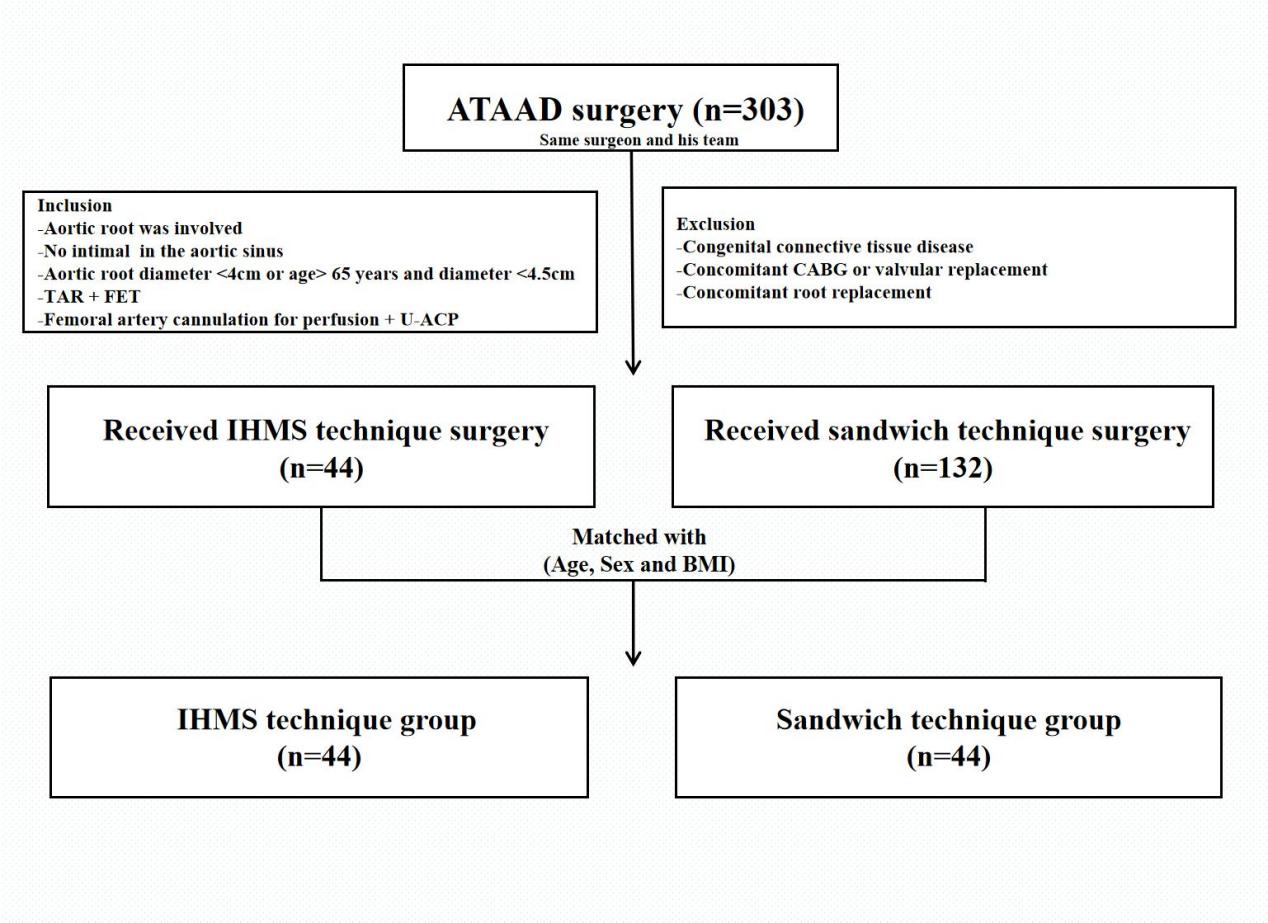


**Fig. S4.** The intimal rupture was located at the sinus-tubular junction.


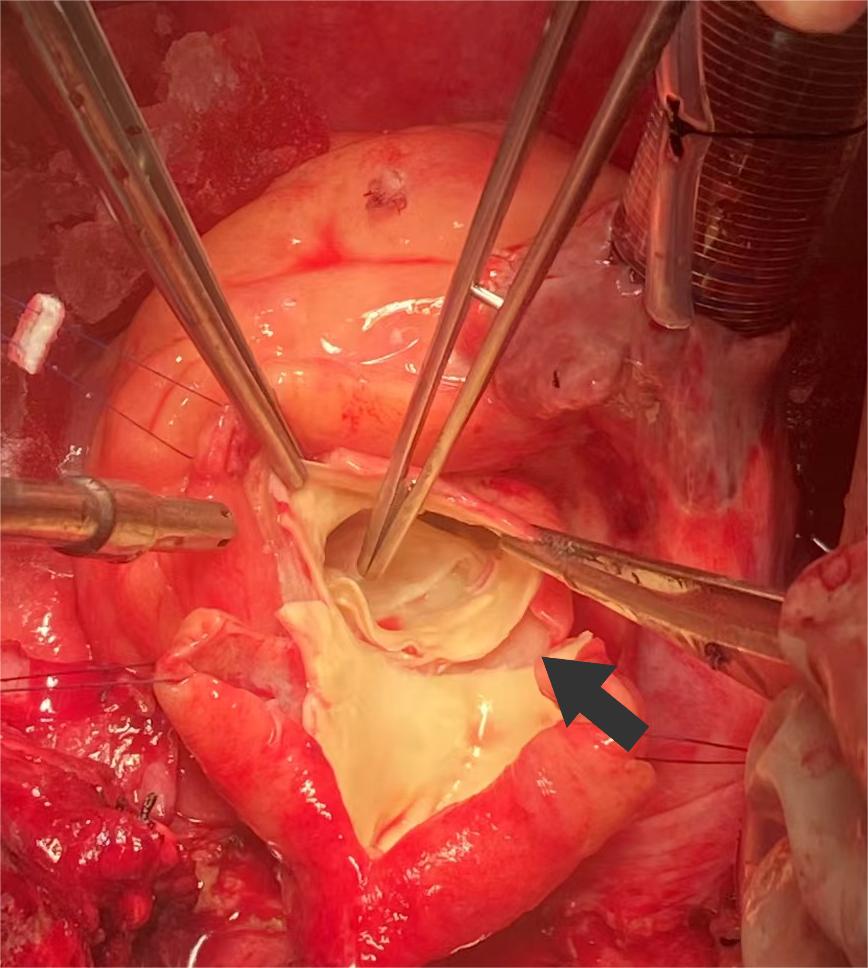

Supplement: Supplemental Information 2 [file peerj-13-19159-s002.docx]
