# Supplementary material for: Intermittent horizontal mattress suture in proximal anastomosis for acute type A aortic dissection: a retrospective study
Source: PeerJ. 2025 Mar 26;13:e19159. doi: 10.7717/peerj.19159 (PMC11954457; doi:10.7717/peerj.19159)
Supplement: Supplemental Information 3 [file peerj-13-19159-s003.docx]

Supplementary table S1. Preoperative and intraoperative characteristics of patients with the intimal tear near STJ

| No. | The intimal tear is within 5mm above the right/left coronary artery origin | Age - years | Gender | Body mass index - kg/m2 | Aortic valve regurgitation | Pericardial tamponade | Operation time - h | Proximal anastomosis time - min | Intraoperative blood loss - L | Spontaneous intraoperative heartbeat recovery |
| --- | --- | --- | --- | --- | --- | --- | --- | --- | --- | --- |
| 1 | Right | 44 | Male | 24.6 | - | - | 4.83 | 56 | 0.8 | - |
| 2 | Right | 48 | Male | 24.5 | - | + | 4.62 | 30 | 1.2 | - |
| 3 | Left | 61 | Male | 24.8 | + | + | 7.23 | 35 | 2 | + |
| 4 | Right | 64 | Male | 20.8 | - | - | 5.03 | 41 | 0.8 | + |
| 5 | Left | 57 | Female | 26.7 | - | - | 6.33 | 33 | 2 | - |
| 6 | Right | 60 | Female | 23.7 | - | - | 5.37 | 32 | 0.8 | + |
| 7 | Left | 52 | Male | 27.7 | - | - | 5.4 | 30 | 0.6 | - |
| 8 | Right | 45 | Male | 27.7 | - | - | 7.68 | 28 | 0.5 | - |

Supplementary table S2. Follow-up characteristics of patients with the intimal tear near STJ

| No. | Hospital stay time - d | Length of ICU stay - d | Re-exploration for bleeding | Aortic valve regurgitation | Aortic Sinus Dilatation | Aortic Root Stenosis | reoperation | current status | follow-up time - month |
| --- | --- | --- | --- | --- | --- | --- | --- | --- | --- |
| 1 | 23 | 11 | - | - | - | - | - | Alive | 23 |
| 2 | 40 | 28 | - | - | - | - | - | Alive | 19 |
| 3 | 24 | 8 | - | - | - | - | - | Alive | 21 |
| 4 | 17 | 9 | - | - | - | - | - | Alive | 20 |
| 5 | 18 | 6 | - | - | - | - | - | Alive | 18 |
| 6 | 17 | 9 | - | - | - | - | - | Alive | 15 |
| 7 | 20 | 13 | - | - | - | - | - | Alive | 12 |
| 8 | 26 | 7 | - | - | - | - | - | Alive | 20 |

ICU = intensive care unit.
