# Supplementary material for: Intermittent horizontal mattress suture in proximal anastomosis for acute type A aortic dissection: a retrospective study
Source: PeerJ. 2025 Mar 26;13:e19159. doi: 10.7717/peerj.19159 (PMC11954457; doi:10.7717/peerj.19159)
Supplement: Supplemental Information 6 [file peerj-13-19159-s006.docx]

The following provides explanations for the categorical variables included in the dataset:

1. Male

- 1: Represents male individuals.

- 0: Represents female individuals.

2. Diabetes Mellitus

- 1: Indicates patients with diabetes mellitus.

- 0: Indicates patients without diabetes mellitus.

3. Hypertension

- 1: Indicates patients with hypertension.

- 0: Indicates patients without hypertension.

4. Cerebral Disease History

- 1: Indicates patients with a history of cerebral disease.

- 0: Indicates patients without a history of cerebral disease.

5. Normal White Blood Cell

- 1: Represents patients with a white blood cell count within the normal range (4.0–10.0) x 10^9/L.

- 0: Represents patients with a white blood cell count outside the normal range.

6. Anemia

- 1: Indicates patients with anemia.

- 0: Indicates patients without anemia.

7. Platelet < 100×10^9/L

- 1: Represents patients with a platelet count below 100×10^9/L.

- 0: Represents patients with a platelet count of 100×10^9/L or above.

8. Elevated INR

- 1: Indicates an INR value greater than 1.5.

- 0: Indicates an INR value of 1.5 or less.

9. Hypoproteinaemia

- 1: Represents patients with albumin levels below 35g/L.

- 0: Represents patients with albumin levels of 35g/L or above.

10. Elevated Triglyceride

- 1: Indicates patients with triglyceride levels exceeding 2.25mmol/L.

- 0: Indicates patients with triglyceride levels not exceeding 2.25mmol/L.

11. Elevated Total Cholesterol

- 1: Represents patients with total cholesterol levels of 6.22mmol/L or above.

- 0: Represents patients with total cholesterol levels below 6.22mmol/L.

12. Elevated Creatinine

- 1: Indicates patients with creatinine levels above the normal range (males > 132μmol/L, females > 111μmol/L).

- 0: Indicates patients with creatinine levels within the normal range.

13. Left Ventricle Ejection Fractions < 50%

- 1: Represents patients with left ventricle ejection fractions below 50%.

- 0: Represents patients with left ventricle ejection fractions of 50% or above.

14. Aortic Valve Regurgitation

- 1: Indicates patients with preoperative aortic valve regurgitation.

- 0: Indicates patients without preoperative aortic valve regurgitation.

15. Transient Ischemic Attack

- 1: Indicates patients with preoperative transient ischemic attack.

- 0: Indicates patients without preoperative transient ischemic attack.

Preoperative Aortic Dissection Involvement

16.Brachiocephalic Trunk, 17.Left Common Carotid Artery, 18. Left Subclavian Artery, 19. Celiac Trunk Artery, 20. Mesenteric Artery, 21. Renal Artery, 22. Lower Extremity Arteries

- 1:Represents patients with preoperative aortic dissection involving the specified artery.

- 0: Represents patients without preoperative aortic dissection involving the specified artery.

23. Pericardial Tamponade

- 1: Indicates patients with preoperative pericardial tamponade.

- 0: Indicates patients without preoperative pericardial tamponade.

24. Spontaneous Heartbeat Recovery

- 1: Represents patients requiring electrical defibrillation for heartbeat recovery.

- 0: Represents patients with spontaneous heartbeat recovery during surgery.

25. Re-exploration for Bleeding

- 1: Indicates patients who underwent re-exploration for bleeding postoperatively.

- 0: Indicates patients who did not undergo re-exploration for bleeding postoperatively.

Postoperative Complications

26.Paraplegia, 27. Stroke, 28. Renal Failure, 29. Pulmonary Infection, 30. Liver Failure, 31. Septicemia, 32. Gastrointestinal Bleeding

- 1:Indicates patients who experienced the specified postoperative complication.

- 0: Indicates patients who did not experience the specified postoperative complication.

33. Advanced Life Support

- 1: Represents patients who required advanced life support postoperatively.

- 0: Represents patients who did not require advanced life support postoperatively.

34. Death (1 Month, 6 Months, 12 Months, 24 Months)

- 1: Indicates patient death within the specified time period.

- 0: Indicates patient survival beyond the specified time period.

35. IHMS (Intermittent horizontal mattress suture)

- 1: Represents the use of the IHMS during surgery for proximal aortic anastomosis.

- 0: Represents the use of the sandwich method during surgery for proximal aortic anastomosis.
