# Supplementary figures and images for: Intermittent horizontal mattress suture in proximal anastomosis for acute type A aortic dissection: a retrospective study
Source: PeerJ. 2025 Mar 26;13:e19159. doi: 10.7717/peerj.19159 (PMC11954457; doi:10.7717/peerj.19159)

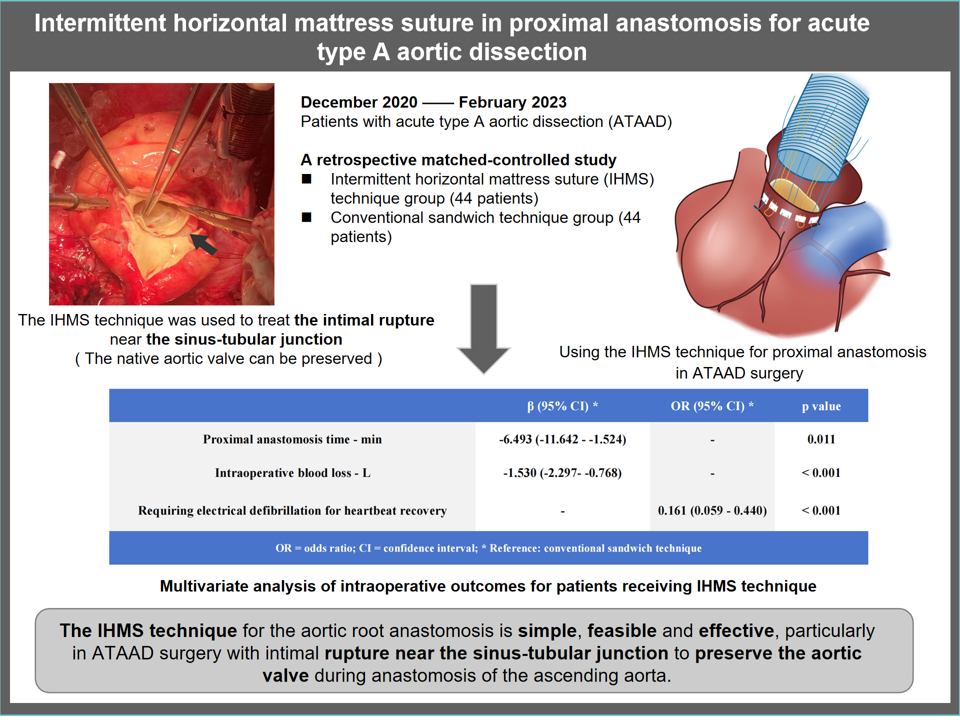

Supplement: Supplemental Information 7 [file peerj-13-19159-s007.png]
